# Association between Hyperreflective Foci on Spectral-Domain Optical Coherence Tomography and Early Recurrence of Diabetic Macular Edema after Intravitreal Dexamethasone Implantation

**DOI:** 10.1155/2019/3459164

**Published:** 2019-11-19

**Authors:** Kyung Tae Kim, Dong Yoon Kim, Ju Byung Chae

**Affiliations:** ^1^Department of Ophthalmology, Gangneung Asan Hospital, College of Medicine, University of Ulsan, Ulsan, Republic of Korea; ^2^Department of Ophthalmology, Chungbuk National University Hospital, College of Medicine, Chungbuk National University, Cheongju, Republic of Korea

## Abstract

**Purpose:**

To investigate the associations between hyperreflective foci (HRF) on spectral-domain optical coherence tomography (SD-OCT) and early recurrence of macular edema after intravitreal dexamethasone (DEX) implantation in eyes with refractory diabetic macular edema (DME) to bevacizumab.

**Methods:**

Medical records of patients with refractory DME to bevacizumab, who underwent intravitreal DEX implantation and 12-month follow-up, were reviewed. Eyes in which central subfield thickness (CST) increased over 50 *μ*m at 3 months compared with the first month after intravitreal DEX implantation were categorized into the early recurrence group, and the others were categorized into the late recurrence group. Best-corrected visual acuity (BCVA), CST, and number of HRF on SD-OCT were analyzed.

**Results:**

Twenty-nine eyes of 26 patients (16 eyes in the early recurrence group and 13 eyes in the late recurrence group) were included in this study. The numbers of HRF in entire retina, inner retina, and outer retina at baseline in the early recurrence group (11.38 ± 3.07 in entire retina, 5.44 ± 1.50 in inner retina, 5.94 ± 2.74 in outer retina) were significantly greater than those in the late recurrence group (7.54 ± 3.60 in entire retina, *p*=0.006; 4.08 ± 1.70 in inner retina, *p*=0.034; 3.46 ± 2.30 in outer retina, *p*=0.013). Multivariate logistic regression analysis showed that a higher number of HRF increased the risk of early recurrence after intravitreal DEX implantation (odds ratio in entire retina: 1.518, *p*=0.012; odds ratio in inner retina: 2.058, *p*=0.027; odds ratio in outer retina: 1.610, *p*=0.029).

**Conclusions:**

Higher baseline numbers of HRF on SD-OCT may be a predictive indicator of early recurrence of macular edema after intravitreal DEX implantation for DME.

## 1. Introduction

Diabetic macular edema (DME), which affects approximately 6.8% of the diabetic population, is the most common cause of visual impairment in patients with diabetic retinopathy [1, 2]. Since vascular endothelial growth factor (VEGF) is an essential endogenous mediator of DME, anti-VEGF injections are effective in improving visual acuity and are generally considered as first-line therapy for DME [3]. Although anti-VEGF injections have become the first-line gold standard treatment for DME, there are many patients who respond poorly to anti-VEGF therapy, and the resolution of fluid is transient and not complete [4–6]. Gonzalez et al. [7] reported that approximately 40% of eyes showed at best only limited improvement in BCVA (<5 letters) after 3 months of anti-VEGF treatment. In patients with poor initial response to anti-VEGF agents, intravitreal steroid injection may be an alternative choice of treatment because inflammation also plays an important role in the pathogenesis of DME [8, 9].

Dexamethasone (DEX) intravitreal implant 0.7 mg (Ozurdex; Allergan, Inc., Irvine, CA, USA) is also a widely used agent for DME because it leads to improvement in visual acuity and decrease in retinal thickness [10, 11], even in eyes with DME that do not respond adequately to anti-VEGF treatment [12–14]. Dexamethasone intravitreal implant is able to release medication for up to 6 months [15, 16]. However, in the live clinical setting, more frequent administration of DEX implant is often required due to the early recurrence of DME. In previous studies, the maximum effects of dexamethasone implant tended to occur at 3 months and to slowly decrease from 4 to 6 months, and the mean interval between DEX injections varied from approximately 3 to 7 months [17–20].

Hyperreflective foci (HRF) on spectral-domain optical coherence tomography (SD-OCT) are well-circumscribed particles that are 20 to 40 *μ*m in diameter and are of equal or higher reflectivity than the retinal pigment epithelium (RPE) band [21, 22]. HRF are known to be associated with extravasation of lipoprotein or increased inflammation in the retina [23–25]. A recent study revealed that DME with no HRF responded better to DEX implants than those with HRF and suggested that HRF can be a functional outcome predictor in DME treated with DEX implant [26]. Another study also reported that the number of HRF on SD-OCT can be a predictive indicator of the treatment response to DEX implant [27]. Based on these reports, we hypothesized that HRF may be associated with an early recurrence of DME and that the greater the number of HRF, the faster the DME might recur after intravitreal DEX implantation. If there was a way to know in advance the early recurrence of DME after intravitreal DEX implantation, retreatment could be initiated in high-risk patients in a timely manner, which probably improves prognosis. Therefore, in our study, we aimed to investigate the association between the number of HRF on SD-OCT and early recurrence of DME after intravitreal DEX implantation.

## 2. Methods

### 2.1. Patient Selection

This was a retrospective, observational, single-center study of consecutive patients who were injected with intravitreal DEX implant as treatment for refractory DME to bevacizumab and followed up for at least 12 months at the Chungbuk National University Hospital, Korea, between March 2015 and June 2017. Inclusion criteria were as follows: (1) refractory DME to bevacizumab and (2) refractory DME completely resolved after intravitreal DEX implantation. Refractory DME was defined as worsening of BCVA by 2 Early Treatment Diabetic Retinopathy Study (ETDRS) lines or reduction of less than 10% of retinal thickness on SD-OCT measured 1 month after more than at least three times anti-VEGF injections that were given at monthly intervals [7]. Exclusion criteria were (1) another concomitant ocular disease that causes macular edema (i.e., neovascular age-related macular degeneration or choroidal neovascularization due to other reasons, retinal vein occlusion, uveitis, and recent intraocular surgery possibly causing postsurgical macular edema); (2) another ocular condition that compromises VA, except for the presence of cataract; and (3) previous treatment with intraocular corticosteroids within the 6 months before treatment with intravitreal DEX implant. Approval of the Institutional Review Board and ethics committees of Chungbuk National University Hospital was granted before the initiation of the study, which was performed in compliance with the tenets of the Declaration of Helsinki.

The eyes were divided into two groups according to recurrence time of macular edema after intravitreal DEX implantation. Eyes in which central subfield thickness (CST) increased over 50 *μ*m at 3 months compared with the first month after intravitreal DEX implantation were categorized into the early recurrence group, and the rest were categorized into the late recurrence group [28].

At the initial visit, all patients underwent a comprehensive ophthalmic examination, including BCVA using a Snellen chart, intraocular pressure (IOP) measurement, slit-lamp examination, color fundus photography, fluorescein angiography, and SD-OCT (Spectralis; Heidelberg Engineering, Heidelberg, Germany). During each visit, ophthalmic examinations, including the assessment of BCVA, applanation tonometry, slit-lamp examination, dilated fundus examination, fundus photography, and SD-OCT, were performed.

Patients charts were reviewed for demographic data, type of diabetic retinopathy, hemoglobin A1c (HbA1c) values, previous treatment for DME, BCVA, and CST before the intravitreal DEX implantation and at 1, 3, 6, and 12 months after the DEX injections.

### 2.2. Optical Coherence Tomography Analysis

For SD-OCT images, a Spectralis OCT (Heidelberg Engineering) was used with a custom 20° × 20° volume acquisition protocol, which consisted of 49 sections. The integrated follow-up mode of the device was used to ensure that the exact same retinal area was imaged at every follow-up visit. CST, ellipsoid zone (EZ) and external limiting membrane (ELM) status, presence of subretinal fluid (SRF), and HRF were assessed and analyzed. The CST was automatically calculated as the average retinal thickness within the central circle of 500 *μ*m radius. Measurements of the disrupted length of the EZ and the ELM were performed within a radius of 1500 *μ*m centered on the fovea-spanning horizontal B-scans. HRF were defined as discrete and well-circumscribed particles, 20 to 40 *μ*m in diameter, and of equal or higher reflectivity than the RPE band on SD-OCT. The number of HRF within an area of 1500 *μ*m radius centered on the fovea on horizontal raster scan was manually counted using the ImageJ software (http://imagej.nih.gov/ij/;provided in the public domain by the National Institutes of Heal, Bethesda, MD, USA). The HRF were subdivided according to the retinal layers: inner retina (between the internal limiting membrane and the inner nuclear layer), outer retina (between the outer plexiform layer and external limiting membrane), and entire retina (between internal limiting membrane and external limiting membrane) [26, 29, 30]. The photoreceptor layer, the RPE, and subretinal space were excluded because the naturally high reflectivity of these layers impedes the evaluation of HRF [30]. Counting and classification were performed by two experienced masked retina specialists (J. B. Chae and D. Y. Kim). In case of disagreement in the counted number of HRF between the two retina specialists exceeded 20%, differences were resolved through discussion. And the average of both investigators was used for analysis.

### 2.3. Statistical Analysis

The SPSS version 22.0 software (SPSS, Inc., Chicago, IL, USA) was used to perform the statistical analyses, and a *p* < 0.05 was considered statistically significant. All values are presented as the means ± SD or numbers (%). The assessment of normality was done initially using the Kolmogorov–Smirnov test.

Student's *t*-test was used to compare quantitative data populations with normal distributions and equal variance. Data were analyzed using the Mann–Whitney *U* test for populations with nonnormal distributions or unequal variance. The comparison between two categorical variables was performed by Fisher's exact test. Multivariate logistic regression analyses were performed to identify the independent baseline factors related to early recurrence. Receiver operation characteristic (ROC) curve analysis was performed to determine the accuracy of prediction regarding the early recurrence of DME.

## 3. Results

Thirty-eight eyes of 36 patients were treated with intravitreal DEX implant for refractory DME to bevacizumab. Finally, 29 eyes of 26 patients (12 men and 14 women) were included in the analysis. The other 9 eyes of 9 patients were excluded from the study for the following reasons: (1) prior history of vitreoretinal surgery (one eye), (2) evidence of retinal disease that might affect visual acuity or macular microstructure (five eyes), and (3) follow-up period less than 12 months (three eyes).

### 3.1. Baseline Characteristics

Patients' demographics and baseline ocular findings are summarized in Table 1. A total of 29 eyes of 26 diabetic patients undergoing intravitreal DEX implantation for refractory DME to bevacizumab were studied. The mean age of the patients was 58.3 ± 9.3 years. The mean HbA1c level was 8.9 ± 1.7%, and 11 patients had concomitant hypertension. Twenty-one eyes (72.4%) had undergone panretinal photocoagulation, and none of the eyes had undergone macular laser photocoagulation. The mean number of prior intravitreal bevacizumab injections was 4.4 ± 2.1 times, and the mean duration of treatment for this mode of therapy was 6.4 ± 4.8 months. Before intravitreal DEX implantation, the mean BCVA was 0.73 ± 0.42 logMAR units and the mean CST was 592.6 ± 180.5 *μ*m.

### 3.2. Early Recurrence and Late Recurrence of DME after Intravitreal DEX Implantation

The eyes were divided into two groups according to timing of recurrence of DME after intravitreal DEX implantation. Of the 29 eyes, 16 eyes (55.2%) were classified as the early recurrence group and 13 eyes as the late recurrence group. The number of prior intravitreal bevacizumab injections was not different between the groups (4.1 ± 1.7 in the early recurrence group, 4.8 ± 2.5 in the late recurrence group). Also, there was no significant difference in duration of treatment for intravitreal bevacizumab injection between the groups (5.3 ± 2.7 in the early recurrence group, 7.9 ± 6.4 in the late recurrence group). The number of HRF on SD-OCT in the inner retina and outer retina was significantly higher in the early recurrence group than in the late recurrence group (*p*=0.034 in inner retina, *p*=0.013 in outer retina). The number of HRF in the entire retina was also significantly higher in the early recurrence group (*p*=0.006). The mean number of HRF in the entire retina before DEX implantation was 11.38 ± 3.07 (5.44 ± 1.50 in inner retina; 5.94 ± 2.74 in outer retina), which decreased significantly at 12 months after DEX implantation to 7.19 ± 2.29 (3.69 ± 1.14 in inner retina, *p* < 0.001; 3.31 ± 2.15 in outer retina, *p* < 0.001) in the early recurrence group. The mean number of HRF in the entire retina before DEX implantation was 7.54 ± 3.60 (4.08 ± 1.70 in inner retina; 3.46 ± 2.30 in outer retina), which decreased significantly at 12 months after DEX implantation to 4.69 ± 3.30 (3.15 ± 1.57 in inner retina, *p*=0.027; 1.31 ± 1.44 in outer retina, *p*=0.001) in the late recurrence group. Other factors on SD-OCT such as CST, EZ, and ELM disruption length did not differ between the two groups (Table 2).

**Figure 1:**
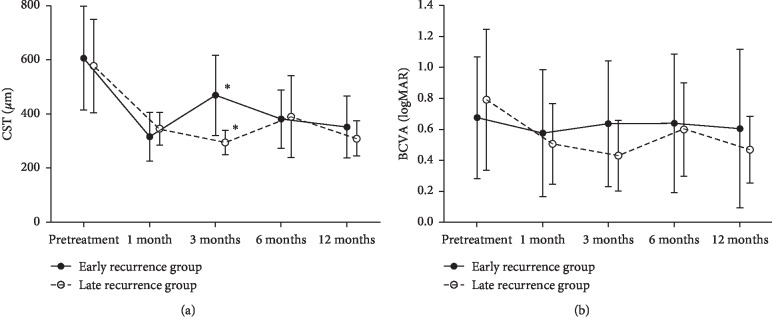
Graph illustrating changes in central subfield thickness (CST) and logarithm of the minimal angle of resolution (logMAR) best-corrected visual acuity (BCVA) at baseline and one, three, six, and 12 months after intravitreal dexamethasone (DEX) implantation for the treatment of refractory diabetic macular edema (DME). (a) The mean CST values before treatment and at 1, 3, 6, and 12 months after intravitreal DEX implantation in early recurrence group were 604.9 ± 191.4, 315.9 ± 90.4, 468.1 ± 148.4, 380.4 ± 108.5, and 351.9 ± 114.6, respectively. In the late recurrence group, the mean CST values were 577.3 ± 172.5, 345.0 ± 60.4, 293.9 ± 45.0, 389.5 ± 151.4, and 309.2 ± 64.2, respectively. The CST values at 3 months (asterisk) between the two groups showed significant difference (*p* < 0.001). (b) The mean BCVA values before treatment and at 1, 3, 6, and 12 months after intravitreal DEX implantation in the early recurrence group were 0.68 ± 0.39, 0.58 ± 0.41, 0.64 ± 0.41, 0.64 ± 0.45, and 0.61 ± 0.51, respectively. In the late recurrence group, the mean BCVA values were 0.79 ± 0.45, 0.51 ± 0.26, 0.43 ± 0.23, 0.60 ± 0.30, and 0.47 ± 0.22, respectively. There were no significant differences of BCVA between the two groups during the follow-up period.

**Figure 2:**
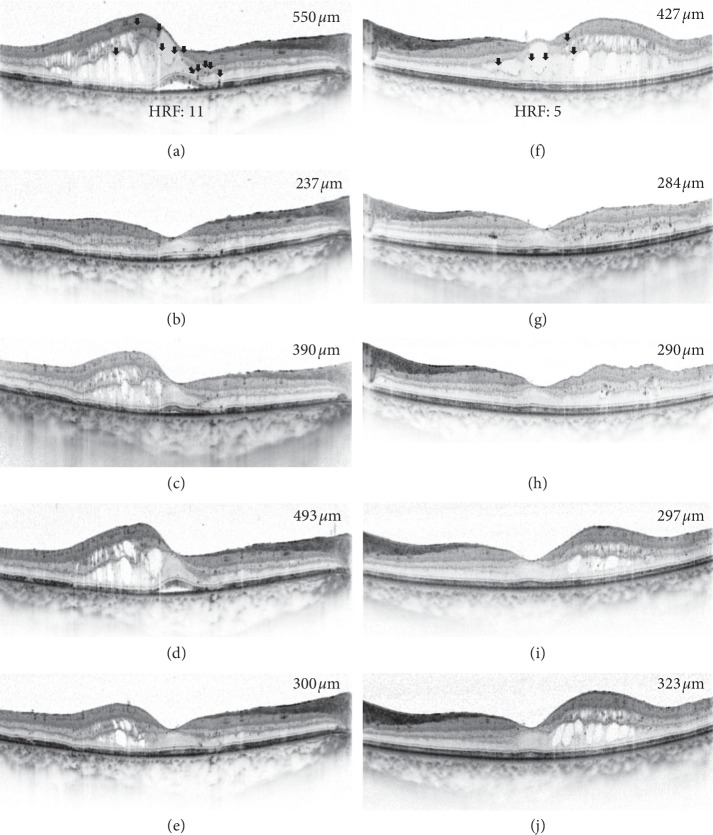
Optical coherence tomography (OCT) scans of representative cases in the early recurrence group and late recurrence group. The left column (A, B, C, D, and E) represents a right eye of a 58-year-old female in the early recurrence group, and she was treated with a total of five bevacizumab injections before intravitreal dexamethasone (DEX) implantation. The right column (F, G, H, I, and J) represents a left eye of a 60-year-old female in the late recurrence group, and she was treated with a total of four bevacizumab injections before intravitreal DEX implantation. The black arrow represents the hyperreflective foci (HRF). (a) Snellen best-corrected visual acuity (BCVA) at baseline was 0.6, and central subfield thickness (CST) at baseline was 550 *μ*m. The number of HRF was 11. (b) The CST was 237 *μ*m at 1 month after intravitreal DEX implantation. (c) The CST increased to 390 *μ*m at 3 months. (d) DEX implant reinjection was performed due to continuous increase of CST for 6 months. The CST was 493 *μ*m at 6 months. (e) The CST had reduced to 300 *μ*m at 12 months. Snellen BCVA was 0.7 at 12 months. (f) Snellen BCVA at baseline was 0.4, and CST at baseline was 427 *μ*m. The number of HRF was 5. (g) The CST was 284 *μ*m at 1 month after intravitreal DEX implantation. (h) The CST was 290 *μ*m at 3 months. (i) The CST was 297 *μ*m at 6 months. (j) The CST had increased to 323 *μ*m, and Snellen BCVA was 0.7 at 12 months.

Changes in mean CST and BCVA after intravitreal DEX implantation are shown in Figure 1. The mean CST values before treatment and at 1, 3, 6, and 12 months after intravitreal DEX implantation in the early recurrence group were 604.9 ± 191.4, 315.9 ± 90.4, 468.1 ± 148.4, 380.4 ± 108.5, and 351.9 ± 114.6, respectively. In the late recurrence group, the mean CST values were 577.3 ± 172.5, 345.0 ± 60.4, 293.9 ± 45.0, 389.5 ± 151.4, and 309.2 ± 64.2, respectively. There was a significant difference in the CST values at 3 months between the two groups (*p* < 0.001). The mean BCVA values before treatment and at 1, 3, 6, and 12 months after intravitreal DEX implantation in the early recurrence group were 0.68 ± 0.39, 0.58 ± 0.41, 0.64 ± 0.41, 0.64 ± 0.45, and 0.61 ± 0.51, respectively. In the late recurrence group, the mean BCVA values were 0.79 ± 0.45, 0.51 ± 0.26, 0.43 ± 0.23, 0.60 ± 0.30, and 0.47 ± 0.22, respectively. There was no significant difference in BCVA between the two groups during the follow-up period. A representative case from each of the two groups is displayed in Figure 2.

In the early recurrence group, 5 eyes were injected with intravitreal anti-VEGF agents (aflibercept in 2 eyes and bevacizumab in 3 eyes) and 2 eyes underwent macular laser photocoagulation as additional treatment for recurrence of DME at 3 months after initial intravitreal DEX implantation. A total of 28 additional treatments (9 times with anti-VEGF injection, 17 times with intravitreal DEX implantation, and 2 times with macular laser photocoagulation) in the early recurrence group were performed during the follow-up period, compared with a total of 10 additional treatments (5 times with anti-VEGF injection and 5 times with intravitreal DEX implantation) in the late recurrence group. There were no significant differences in BCVA and CST between the two groups at 12 months.

### 3.3. Relation of Early Recurrence of DME with Clinical Data

Logistic regression analyses were performed to identify the baseline risk factors in the eyes with early recurrence of DME after intravitreal DEX implantation (Table 3). The result of univariate logistic regression analysis showed that the numbers of HRF in the inner, outer, and entire retina, respectively, were significant risk factors for early recurrence of DME (odds ratio in inner retina: 1.712, *p*=0.041; odds ratio in outer retina: 1.578, *p*=0.030; odds ratio in entire retina: 1.424, *p*=0.014). According to the multivariate regression analysis with adjustments for age and sex, the numbers of HRF in the inner, outer, and entire retina were also associated with early recurrence of DME after intravitreal DEX implantation (odds ratio in inner retina: 2.058, *p*=0.027; odds ratio in outer retina: 1.610, *p*=0.029; odds ratio in entire retina: 1.518, *p*=0.012).

**Figure 3:**
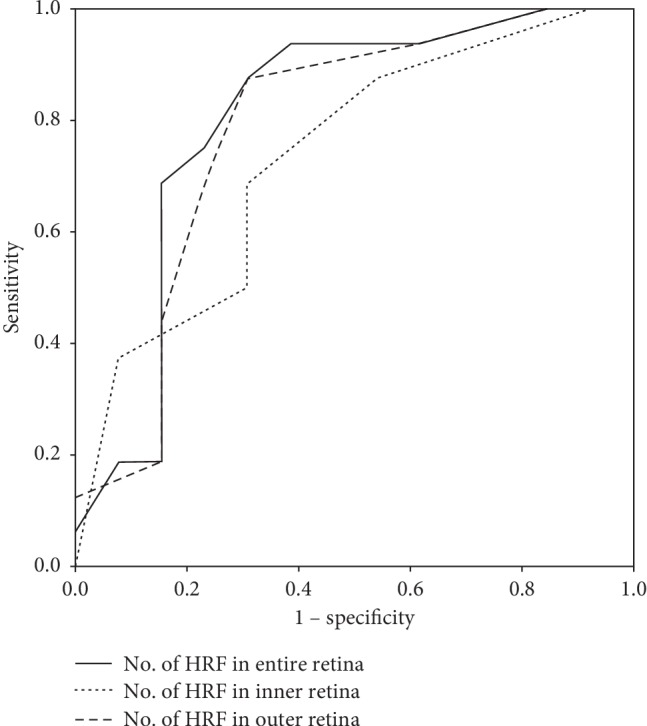
ROC curves for HRF showing early recurrence of diabetic macular edema (DME) after intravitreal dexamethasone (DEX) implantation. To evaluate the predictive accuracy of the number of HRF for early recurrence of DME, ROC curve analysis was performed. The areas under the ROC curve (AUROC) for the number of HRF in the inner, outer, and entire retina were 0.733 (0.547–0.919; *p*=0.033), 0.784 (0.601–0.966; *p*=0.010), and 0.805 (0.627–0.983; *p*=0.005), respectively. The number of HRF in entire retina had a cutoff value of 9.50, with a sensitivity and specificity of 0.750 and 0.769, respectively.

To evaluate the accuracy of the number of HRF in the prediction of early recurrence of DME, ROC curve analysis was performed (Figure 3). The areas under the ROC curve (AUROC) for the number of HRF in the inner, outer, and entire retina were 0.733 (0.547–0.919; *p*=0.033), 0.784 (0.601–0.966; *p*=0.010), and 0.805 (0.627–0.983; *p*=0.005), respectively. The number of HRF in entire retina had a cutoff value of 9.50, with a sensitivity and specificity of 0.750 and 0.769, respectively.

### 3.4. Adverse Events

Four of 8 phakic eyes in the early recurrence group and 4 of 9 phakic eyes in the late recurrence group underwent cataract surgery between 6 months and 12 months after intravitreal DEX implantation because of cataract development or progression. Additionally, 15 of 29 eyes (8 of 16 eyes in the early recurrence group and 7 of 13 eyes in the late recurrence group) required antiglaucoma medication because of increased IOP higher than 21 mmHg after intravitreal DEX implantation. One eye of each group was still receiving antiglaucoma medication at the last follow-up. In all cases, IOP increased within three months after intravitreal DEX implantation and was successfully treated with antiglaucoma medication. None of the patients required trabeculectomy or other filtering surgery to control IOP. Furthermore, there were no severe postoperative complications such as retinal detachment, iris neovascularization, malposition of DEX implant, or endophthalmitis after intravitreal DEX implantation.

## 4. Discussion

One randomized clinical trial reported that patients with DME achieved functional and anatomical improvement with an average of only 4 to 5 DEX injections over 3 years [10]. Other results from surveys aiming to monitor the real dispensing of drugs through physicians, pharmacies, and social security showed that the average DEX implant injection were 2.4 per year with a time-window between treatment ranging between 4.7 and 5.2 months [31]. The interval between DEX injections varied from approximately 3 to 7 months in previous studies [17–20]. Based on these results, it can be seen that the timing of retreatment after intravitreal DEX implantation may vary between patients. However, there is few reliable data that shed light on the duration of the effect of DEX implant in individual patients. Therefore, we analyzed the timing of recurrence of DME through HRF in this study. The primary finding of this study was that the number of HRF in the early recurrence group was significantly more than that in the late recurrence group. A large number of HRF at baseline was a risk factor for early recurrence of DME. Therefore, the principle message of this study is that the number of HRF may be a predictive factor of early recurrence of DME after intravitreal DEX implantation. This message could be of importance for clinical practice, because we can infer in advance the possibility of early recurrence of DME after DEX injection based on the number of HRF at baseline, thereby getting a hint to decide the timing of follow-up or retreatment.

Early recurrence was defined as increasing CST over 50 *μ*m at 3 months compared with the first month after intravitreal DEX implantation in this study. Visual function is the most relevant outcome measure, but it is a subjective measure of treatment response and can be influenced by cataract progression, one of the side effects of DEX implant. Actually, a total of 8 eyes underwent cataract surgery between 6 months and 12 months after DEX injection because of cataract development or progression in this study. Conversely, anatomical measurements such as CST on SD-OCT are more objective and reliable outcome measures for treatment response. For this reason, we defined early recurrence using CST except for BCVA.

In this study, the mean CST was significantly higher at 3 months after DEX implant in the early recurrence group, and the mean BCVA did not differ to a statistically significant degree but was worse in the early recurrence group than in the late recurrence group. Additional treatments were performed from 3 months after first DEX implantation in the early recurrence group and from 5 months in the late recurrence group by intravitreal anti-VEGF injection, macular laser photocoagulation, or DEX implant for treatment of recurrent DME. And the total number of additional treatments required was higher in the early recurrence group than in the late recurrence group. The differences in the mean CST and BCVA between the two groups were gradually reduced with additional treatment, and there were no significant differences in BCVA and CST between the two groups at 12 months. These results were probably due to earlier and more additional treatments in the early recurrence group. These observations suggest that frequent follow-up and active additional treatment should be considered in DME with a large number of HRF after intravitreal DEX implantation.

Several theories have attempted to explain the pathophysiology of HRF, but their precise nature remains unclear. Bolz et al. [23] reported that HRF are the morphologic manifestations of lipoprotein extravasation in DME. They suggested that the HRF represent lipoproteins and/or proteins that have been extruded from the vascular compartment and are a very early subclinical sign of breakdown of the blood-retinal barrier (BRB) in DME [23]. Other studies have stated that HRF are associated with inflammatory responses in the retina [32–35]. As the retinal inflammation increases, microglial cells are transformed into an activated state, increasing in number and translocating through the retina [35]. When microglial cells are activated, their morphology changes and they aggregate [32]. These activated and aggregated microglial cells appear as HRF on SD-OCT [34]. Therefore, an increased number of HRF on SD-OCT may indicate an activated inflammatory process in the retina. The inference from the above studies might be that a large number of HRF reflects severe damage of inner BRB or severe retinal inflammation. Since the breakdown of the BRB and inflammatory reaction play an important role in the pathogenesis of DME [8, 9], DME may be more likely to be present in patients with a large number of HRF. Also, this suggests that a large number of HRF may be associated with early recurrence of DME after treatment. In our study, the numbers of HRF in the retina at baseline in the early recurrence group were significantly greater than those in the late recurrence group. In addition, a large number of HRF showed correlation with early recurrence of DME after intravitreal DEX implantation.

A relationship between HRF and anatomical treatment response according to therapeutic agents for DME has not been established clearly. In patients with DME treated with anti-VEGF, Schreur et al. [30] reported that higher baseline numbers of HRF have predictive value for treatment response in terms of visual acuity improvement and CST decrease after 3 months. Contrarily, Hwang et al. [27] reported that fewer numbers of HRF were associated with good CST response after 3 months of bevacizumab treatment. Similarly, in case of DEX implant, one study stated that eyes with no HRF at baseline were more likely to show good response in CST at 4 months [26], but another study reported that more HRF were observed in good responders to DEX implant [27]. In our study, the degree of CST improvement after 3 months in the early recurrence group was smaller than that in the late recurrence group. The reason for this disparity between studies may be due to differences in study design such as inclusion criteria, definition of HRF, and definition of treatment response.

Several studies reported that HRF correlated negatively with visual acuity [21, 36]. Uji et al. [36] showed that the presence of HRF in the outer retina is closely associated with disrupted ELM and the inner and outer segment (IS/OS) line on SD-OCT images and reduced BCVA in DME. One study reported that HRF are associated with poorer visual outcome in patients with macular edema due to retinal vascular diseases after intravitreal dexamethasone or ranibizumab [37], and another study also reported that preoperative HRF in the outer retinal layers on SD-OCT might predict damage to photoreceptors and a poorer prognosis after vitrectomy for DME [38]. The pathologic association of HRF with disruption of the outer retina and poor visual acuity suggests that these are clinical markers of outer BRB and consequent photoreceptor dysfunction [37]. Unlike the above studies, although difference of EZ disruption length between the early recurrence group and the late recurrence group was almost statistically significant, there were no significant differences between the two groups in BCVA, ELM, and EZ disruption length in our study. Analyzing patients with refractory DME to bevacizumab, not naïve patients, and small sample size may be the reasons for the difference in the present study.

The strength of the current study is that it provides new insights into the association between HRF on SD-OCT and early recurrence of ME in eyes with DME. However, our study had some notable limitations that were inherent in its retrospective and nonrandomized design. And the manual measurement and classification of the position of the HRF may have introduced a subjective element. Although two masked retinal specialists performed the counting, this method inevitably resulted in counting errors. In addition, the sample was relatively small, and we did not include the treatment naïve patients with DME to investigate correlation between the number of HRF and early recurrence. In our hospital, anti-VEGF agents (especially bevacizumab) was first used for DME treatment and it was changed to DEX implant if the effect of anti-VEGF was suboptimal, so the number of naïve patients was relatively small and only DME patients refractory to bevacizumab were included. Therefore, future large-scale prospective studies including treatment naïve DME patients with an automatic quantification system for HRF are warranted.

In conclusion, this study demonstrated that the number of HRF on SD-OCT in patients with refractory DME to bevacizumab can be a potential predictive indicator of the early recurrence of DME after intravitreal DEX implantation. And similar results in BCVA and CST were observed with additional treatment even in the early recurrence group. Therefore, in patients with DME, if a large number of HRF are observed before treatment, the possibility of early recurrence of ME after DEX implant should be considered and additional treatment should be administered after frequent follow-up consultations.

## Figures and Tables

**Table 1:** Patient's demographics and baseline ocular findings.

Characteristics	Value
No. of patients	26
No. of eyes	29
Age, mean ± SD (years)	58.3 ± 9.3
Sex, male/female (%)	12/14 (46/54)
Type of diabetes, type 1/type 2 (%)	2/24 (8/92)
Hemoglobin A_1C_, mean ± SD (%)	8.9 ± 1.7
Hypertension (%)	11 (42)
Type of diabetic retinopathy, NPDR/PDR (%)	18/11 (62/38)
Lens status, phakic/pseudophakic (%)	17/12 (59/41)
Panretinal photocoagulation (%)	21 (72)
No. of prior intravitreal bevacizumab injections, mean ± SD	4.4 ± 2.1
Duration of treatment for intravitreal bevacizumab injection, mean ± SD (month)	6.4 ± 4.8
Best-corrected visual acuity (log MAR), mean ± SD	0.73 ± 0.42
Central subfield thickness, mean ± SD (*μ*m)	592.6 ± 180.5

SD = standard deviation; NPDR = nonproliferative diabetic retinopathy; PDR = proliferative retinopathy; logMAR = logarithm of the minimal angle of resolution.

**Table 2:** Clinical and OCT findings between the early recurrence group and late recurrence group.

Characteristics	Early recurrence group (*n* = 16)	Late recurrence group (*n* = 13)	*p* value
Age, mean ± SD (year)	60.2 ± 10.7	56.0 ± 6.8	0.233^*∗*^
Sex, male/female (%)	6/9 (40/60)	6/5 (55/45)	0.467^‡^
Type of diabetes, type1/type 2 (%)	0/16 (0/100)	2/11 (15/85)	0.104^‡^
Hemoglobin A_1C_, mean ± SD (%)	9.0 ± 1.7	8.2 ± 1.7	0.300^*∗*^
Hypertension (%)	6 (40)	5 (45)	0.781^‡^
Type of diabetic retinopathy, NPDR/PDR (%)	11/5 (69/31)	7/6 (54/46)	0.274^‡^
Lens status, phakic/pseudophakic (%)	8/8 (50/50)	9/4 (69/31)	0.451^‡^
Panretinal photocoagulation (%)	11 (69)	10 (77)	0.697^‡^
No. of prior intravitreal bevacizumab injections, mean ± SD	4.1 ± 1.7	4.8 ± 2.5	0.417^†^
Duration of treatment for intravitreal bevacizumab injection, mean ± SD (months)	5.3 ± 2.7	7.9 ± 6.4	0.190^*∗*^
Best-corrected visual acuity (log MAR), mean ± SD	0.68 ± 0.39	0.79 ± 0.45	0.464^*∗*^
Central subfield thickness, mean ± SD (*μ*m)	604.9 ± 191.4	577.3 ± 172.5	0.690^*∗*^
Subretinal fluid	5 (31)	1 (8)	0.119^‡^
No. of HRF, mean ± SD			
Entire retina	11.38 ± 3.07	7.54 ± 3.60	0.006^*∗*^
Inner retina	5.44 ± 1.50	4.08 ± 1.70	0.034^*∗*^
Outer retina	5.94 ± 2.74	3.46 ± 2.30	0.013^*∗*^
ELM disruption length, mean ± SD (*μ*m)	86.20 ± 69.28	78.46 ± 108.23	0.440^†^
EZ disruption length, mean ± SD (*μ*m)	268.87 ± 77.34	199.00 ± 132.16	0.072^†^

SD = standard deviation; PDR = proliferative retinopathy; logMAR = logarithm of the minimal angle of resolution; HRF = hyperreflective foci; ELM = external limiting membrane; EZ = ellipsoid zone. ^*∗*^*P* values for Student's *t*-test. ^†^*P* values for the Mann–Whitney *U* test. ^‡^*P* values for Fisher's exact test.

**Table 3:** Logistic regression analyses of risk factors associated with early recurrence of DME.

Variables (*n* = 29)	Univariate logistic regression		Multivariate logistic regression					
			HRF in entire retina		HRF in inner retina		HRF in outer retina	
	Odds ratio (95% confidence interval)	*p*	Odds ratio (95% confidence interval)	*p*	Odds ratio (95% confidence interval)	*p*	Odds ratio (95% confidence interval)	*p*
Age	0.052 (0.965–1.155)	0.268	1.056 (0.960–1.162)	0.264	1.056 (0.959–1.163)	0.271	1.051 (0.957–1.154)	0.302
Sex, male	1.667 (0.116–2.277)	0.323	0.424 (0.056–3.223)	0.407	0.342 (0.047–2.477)	0.288	0.662 (0.105–4.180)	0.660
BCVA, logMAR	0.496 (0.080–3.091)	0.453	—	—	—	—	—	—
Hemoglobin A_1C_	1.322 (0.790–2.213)	0.289	—	—	—	—	—	—
PDR	0.390 (0.085–1.779)	0.224	—	—	—	—	—	—
Duration of treatment for intravitreal bevacizumab injection	0.876 (0.724–1.060)	0.174	—	—	—	—	—	—
CST	1.001 (0.997–1.005)	0.678	—	—	—	—	—	—
SRF	5.455 (0.548–54.276)	0.148	—	—	—	—	—	—
No. of HRF in entire retina	1.424 (1.076–1.886)	0.014	1.518 (1.095–2.103)	0.012	—	—	—	—
No. of HRF in inner retina	1.712 (1.022–2.866)	0.041	—	—	2.058 (1.087–3.897)	0.027	—	—
No. of HRF in outer retina	1.578 (1.046–2.380)	0.030	—	—	—	—	1.610 (1.049–2.470)	0.029
ELM disruption length	1.001 (0.992–1.010)	0.813	—	—	—	—	—	—
EZ disruption length	1.007 (0.998–1.016)	0.117	—	—	—	—	—	—

DME = diabetic macular edema; HRF = hyperreflective foci; BCVA = best-corrected visual acuity; logMAR = logarithm of the minimal angle of resolution; PDR = proliferative retinopathy; CST  =  central subfield thickness; SRF = subretinal fluid; ELM = external limiting membrane; EZ = ellipsoid zone.

## Data Availability

The data used to support the findings of this study are restricted by the Institutional Review Board of Chungbuk National University Hospital in order to protect patient privacy. Data are available from Ju Byung Chae (jbchae@chungbuk.ac.kr) for researchers who meet the criteria for access to confidential data.
